# Structural and Visible-Near Infrared Optical Properties of Cr-Doped TiO_2_ for Colored Cool Pigments

**DOI:** 10.1186/s11671-017-2365-5

**Published:** 2017-11-17

**Authors:** Le Yuan, Xiaolong Weng, Ming Zhou, Qingyong Zhang, Longjiang Deng

**Affiliations:** 1 National Engineering Research Center of Electromagnetic Radiation Control Materials, University of Electronic Science and Technology of China, Chengdu, 610054 People’s Republic of China; 2 Key Laboratory of Fluid and Power Machinery of Ministry of Education, Center for Advanced Materials and Energy, Xihua University, Chengdu, 610039 People’s Republic of China

**Keywords:** Titanium dioxide, Cr-doping, Reflection spectrum, Color, Cool pigment

## Abstract

Chromium-doped TiO_2_ pigments were synthesized via a solid-state reaction method and studied with X-ray diffraction, SEM, XPS, and UV-VIS-NIR reflectance spectroscopy. The incorporation of Cr^3+^ accelerates the transition from the anatase phase to the rutile phase and compresses the crystal lattice. Moreover, the particle morphology, energy gap, and reflectance spectrum of Cr-doped TiO_2_ pigments is affected by the crystal structure and doping concentration. For the rutile samples, some of the Cr^3+^ ions are oxidized to Cr^4+^ after sintering at a high temperature, which leads to a strong near-infrared absorption band due to the ^3^A_2_ → ^3^ T_1_ electric dipole-allowed transitions of Cr^4+^. And the decrease of the band gap causes an obvious redshift of the optical absorption edges as the doping concentration increases. Thus, the VIS and near-infrared average reflectance of the rutile Ti_1 − *x*_Cr_*x*_O_2_ sample decrease by 60.2 and 58%, respectively, when the Cr content increases to *x* = 0.0375. Meanwhile, the color changes to black brown. However, for the anatase Ti_1 − *x*_Cr_*x*_O_2_ pigments, only the VIS reflection spectrum is inhibited by forming some characteristic visible light absorption peaks of Cr^3+^. The morphology, band gap, and NIR reflectance are not significantly affected. Finally, a Cr-doped anatase TiO_2_ pigment with a brownish-yellow color and 90% near-infrared reflectance can be obtained.

## Background

TiO_2_ is an important cool pigment applied widely in energy-efficient buildings due to its high visible light (VIS) and near-infrared (NIR) reflectance (> 85%) [1, 2]. Since the sunlight in visible light and near-infrared waveband plays the most important role in heat generation [3, 4], heat-reflective paints prepared by TiO_2_ pigments can obviously decrease the heat accumulation of buildings. This results in a decrease of more than 20% in energy consumption for air conditioning [4]. However, because of the high VIS reflectance of TiO_2_ pigment, the resulting white paint is very bright and unpleasant to the human eye. This also leads to poor esthetics, low stain resistance, and a short lifespan [5, 6]. To overcome these limitations, numerous efforts have been made to develop a novel non-white cool pigment with low lightness and low VIS reflectance while retaining the high NIR reflectance. However, it is difficult to appropriately control the VIS and NIR reflection spectrum simultaneously.

Elemental doping is an effective VIS spectral controlling method that is widely used in many fields, including photo catalysis, photoluminescence, and ceramic pigments [7–9]. For oxide pigment, the doped ions are helpful in forming impurity levels, reducing the band gap, and increasing the ability to absorb low-energy photons, such as the diffuse reflectance spectra of the doped TiO_2_ that can be significantly shifted to longer wavelengths with enhanced visible absorption [10–12]. Hence, it can be used to prepare various colored pigments, such as orange (doping Cr element), tan (Mn), yellow (Ni), and gray (V) [9, 10].

In addition to enhancing the visible light absorption, doped ions further influence the concentrations of the free carriers. Because free carrier absorption is the main photon absorption mechanism in the NIR region, the NIR reflectance of oxide pigments can be improved by controlling the concentrations of free carriers. In addition, the NIR reflectance is also connected to the TiO_2_ host material properties, such as crystal structure, particle morphology, and size. In view of the different mechanisms influencing VIS and NIR reflectance, doped TiO_2_ pigments should be able to be prepared with dark color and high NIR reflectance. This would simultaneously satisfy the need for energy savings and a pleasing color palette.

The aim of this work is to explore the applicability of Cr-doped TiO_2_ as a colored cool pigment. Several samples with different Cr-doped concentrations and sintering temperatures were synthesized via a solid-state reaction method. The influences on crystalline phase, morphology, chemical components, color, and the VIS-NIR reflection spectrum were systematically investigated.

## Experimental

### Synthesis of Ti_1 − *x*_Cr_*x*_O_2_ Pigment

In a typical solid-state reaction process of Ti_1 − *x*_Cr_*x*_O_2_ samples, stoichiometric commercial-grade raw materials of TiO_2_ (99.9%) and Cr_2_O_3_ (99.9%) were milled using a planetary ball mill for 4 h at 450 rpm in ethanol. Agate jar and balls were used. The weight of the mixed powder sample was 50 g, and the ratio of ball weight to sample weight was 10:1. Residual ethanol was removed by evaporation drying about 80 °C. The grinded powders were then calcined at temperature of 800–1000 °C for 4 h in air atmosphere at the heating rate of 5 °C/min. The ensuing pigment powders were ground in agate mortar.

### Characterization

The samples were characterized by X-ray diffraction (D2 PHASER with CuKa radiation, Bruker) and field emission scanning electron microscopy (QUANTA 250, FEI). The lattice constants were calculated from the XRD patterns using the MDI Jade software package. X-ray photoelectron spectroscopy with Al Kα X-ray (h*ν* = 1486.6 eV) radiation operated at 150 W (Thermo Scientific Escalab 250Xi, USA) was used to investigate the surface properties. The shift of the binding energy due to the relative surface charging was corrected using the C 1s level at 284.8 eV as an internal standard. The UV-VIS-NIR reflection spectrum (250-2500 nm) was measured with a UV-VIS-NIR spectrophotometer (Lambda 750, Perkin-Elmer). The CIE LAB color data (*L* ^*^, *a* ^*^, and *b* ^*^) were calculated from the visible light reflection spectrum by Color CIE software (Perkin-Elmer, CIE D65 photo source, and 10° observation angle; the calculated spectrum range was 400–700 nm). And the band gap *E*_*g*_ of powder samples was extracted via the following equation [13, 14]:

1 $$ \left\{\begin{array}{c}{\left[F(R) h\nu \right]}^2=C\left( h\nu -{E}_g\right)\\ {}F(R)=\frac{{\left(1-R\right)}^2}{2R}\end{array}\right. $$

where *F*(*R*) is the Kubelka-Munk function, *R* is the diffuse reflectance, *hν* is the photon energy, and *C* is the proportionality constant.

## Result and Discussion

### Phase Structure of the Samples

**Fig. 1:**
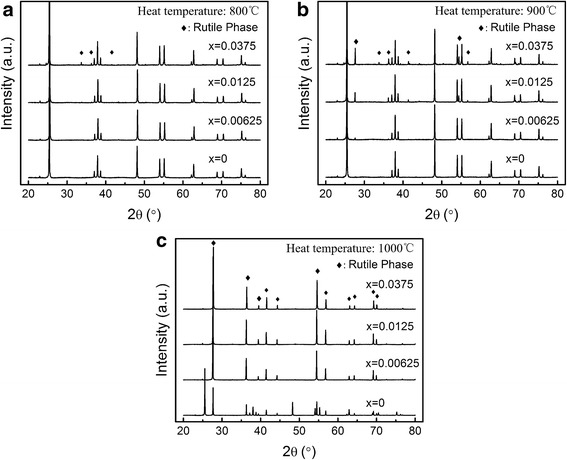
**a**–**c** XRD patterns of Ti_1 − *x*_Cr_*x*_O_2_ products prepared at different sintering temperatures and doping concentrations (Sintering temperature is **a**: 800°C; **b**: 900°C; **c**: 1000°C;)

The XRD patterns of Ti_1 − *x*_Cr_*x*_O_2_ powders with various Cr-doped concentrations obtained at different sintering temperatures from 800 °C to 1000 °C are shown in Fig. 1. The samples calcined at 800 °C have only diffraction peaks of the anatase phase (JCPDS, File No. 21-1272). Traces of the diffraction peaks of the rutile phase (JCPDS, File No. 21-1276) can be found until the doping concentration reaches *x* = 0.0375.

When the sintering temperature is 900 °C (Fig. 1b), the undoped TiO_2_ sample (*x* = 0) has only an anatase crystal structure. It began to transform to the rutile phase as the Cr^3+^ ions are doped into the TiO_2_ matrix. Furthermore, the rutile phase continuously increases with the increasing Cr^3+^ concentration. With the continued increase in sintering temperature to 1000 °C (XRD data; Fig. 1c), there are both the anatase and rutile phases of TiO_2_ in the undoped product. However, the anatase peaks are not detected in Ti_1 − *x*_Cr_*x*_O_2_ products. This illustrates that the Cr^3+^ ions accelerate the crystal phase transformation from anatase to rutile and the phase transition temperature can be reduced by around 100 °C. This is because when the valence (III) cations diffuse in the titania lattice, they provide a charge compensation process to form oxygen vacancies that enhance the transport of atoms and accelerate the anatase-to-rutile phase transition [15, 16].

The Ti_1 − x_Cr_x_O_2_ products calcined at 800 ~ 1000 °C have no chromium oxide diffraction peaks in XRD, which indicates that the Cr dopants are well-dispersed on the TiO_2_ matrix. In addition, the lattice constant of the Ti_1 − *x*_Cr_*x*_O_2_ products is also affected by the concentration of Cr^3+^ impurities (Table 1). Although Cr^3+^ has a slightly larger size (75.5 pm) than Ti^4+^ (74.5 pm), the lattice constant of Ti_1 − *x*_Cr_*x*_O_2_ products decreases with increasing Cr^3+^ concentration regardless of anatase or rutile structure. This might be because of the oxygen vacancy that is formed when Ti–O breaks and Cr^3+^ substitutes into the Ti^4+^ lattice sites [17]. Higher Cr^3+^ concentrations result in more oxygen vacancies. An oxygen deficiency could diminish the number of Ti–O or Cr–O bonds, and this leads to contraction of the O–Ti–O or O–Cr–O bond angle [17]. On the other hand, some Cr^3+^ is gradually oxidized to the smaller Cr^4+^ (55 pm) during the high-temperature sintering process. The overall result is a squeezing of the lattice and a reduction in the values of the lattice constant.

**Table 1:** Variation in the lattice constant with Cr concentrations

The impurity concentration	Lattice constant					
	800 °C (anatase)			1000 °C (rutile)		
	*a* = *b* (*Å*)	*c* (*Å*)	Vol (*Å* ^3^)	*a* = *b* (*Å*)	*c* (*Å*)	Vol (*Å* ^3^)
*x* = 0	3.778	9.499	135.56	–	–	–
*x* = 0.00625	3.771	9.486	134.89	4.575	2.952	61.79
*x* = 0.0125	3.769	9.483	134.74	4.573	2.952	61.73
*x* = 0.025	3.768	9.480	134.56	4.571	2.948	61.6
*x* = 0.0375	3.767	9.476	134.47	4.566	2.946	61.41

### Sample Morphology

**Fig. 2:**
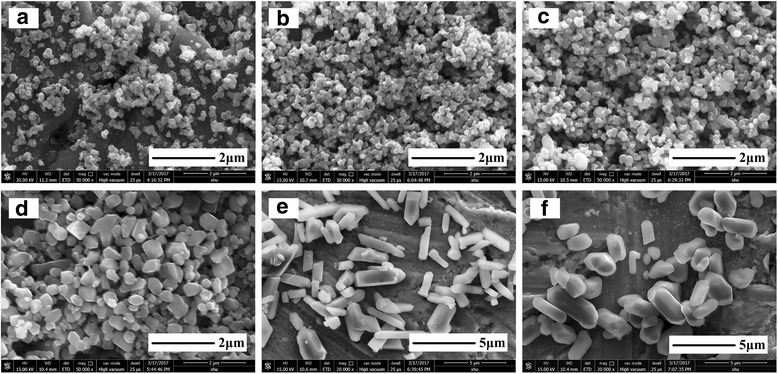
SEM photographs of undoped TiO_2_ and Ti_1 − *x*_Cr_*x*_O_2_ powders: **a** undoped TiO_2_, 800 °C; **b** *x* = 0.00625, 800 °C; **c** *x* = 0.0375, 800 °C; **d** undoped TiO_2_, 1000 °C; **e** *x* = 0.00625, 1000 °C; and **f** *x* = 0.0375, 1000 °C

Figure 2 shows SEM images of undoped TiO_2_ and Ti_1 − *x*_Cr_*x*_O_2_ products prepared at different sintering temperatures and Cr concentrations. The morphology of undoped TiO_2_ samples sintered at 800 °C are nearly spherical, and the average particle size is less than 100 nm. The morphology and particle size have no obvious change upon doping low concentrations of Cr^3+^ (*x* = 0.00625). However, if the doping concentration of Cr^3+^ is too high (*x* = 0.0375), then the particle size would slightly increase, and the morphology becomes non-uniform.

When the temperature increases to 1000 °C, nearly spherical and nearly cubic particles are observed simultaneously in the undoped samples (Fig. 2d) due to the coexistence of anatase and rutile structures. The particle morphology changes to the elongated columnar shape after the Cr^3+^ dopant is added. However, the aspect ratio decreases, and particle size increases with increasing dopant content. There is a tendency to return to a spherical particle again at high doping concentrations. As the doping amount increases to *x* = 0.0375 relative to the undoped sample, the average particle size increases from 300 nm to 2 μm.

### XPS Analysis

**Fig. 3:**
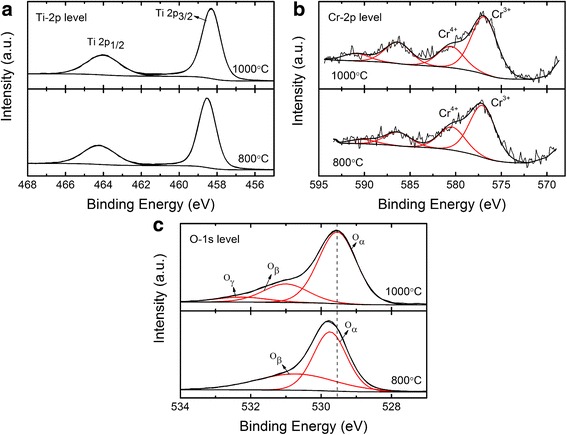
XPS spectra of the **a** Ti-2*p*, **b** Cr-2*p*, and **c** O-1*s* level in Ti_1 − *x*_Cr_*x*_O_2_ samples (*x* = 0.00625)

The XPS spectrum of Cr-doped TiO_2_ powders reveals Cr, Ti, and O. The Ti 2p XPS spectra are presented in Fig. 3a. The results show that there are two major peaks located near 458.9 to 458.3 eV and 464.2 to 464.1 eV. The locations of the major peaks represent the Ti 2p_1/2_ and Ti 2p_3/2_ orbit, respectively, indicating that the Ti element mainly exists as a chemical state of Ti^4+^ [11].

Figure 3-b indicates that all of the samples have two pronounced Cr-2*p* XPS peaks with binding energies of 577 eV and 586.4 eV, which are consistent with the values of Cr^3+^ in the TiO_2_ lattices [18]. The other peaks are located at 580.6 eV and 591 eV, and these are attributed to Cr^4+^ ions [18]. Meanwhile, the area ratios of the Cr^4+^ peak at 580.6 eV increases from 29.6% to 35.8% with annealing temperatures that increase from 800 °C to 1000 °C. Tetravalent Cr^4+^ has been reported to form via a charge compensation reaction triggered by evaporation of Cr [18]. The relative content of Cr^4+^ increases as the annealing temperature increases because the evaporation could be enhanced at a high temperature.

The XPS spectra of O 1s are shown in Fig. 3c. For the sample sintered at 800 °C, the O 1s peaks comprise two overlapping peaks, indicating the existence of different types of oxygen on the samples’ surface. The lower binding energy peak at 529.8 eV is attributed to the lattice oxygen (O_α_) [19]. The other overlapping peak at a binding energy of 530.8 is attributed to surface adsorbed oxygen (O_β_). Specifically, a new overlapping peak is formed at 532.3 eV due to the surface oxygen of hydroxyl or absorbed water (O_γ_) as the annealing temperature increased from 800 to 1000 °C [19]. Moreover, the binding energy of the O 1s peaks tends to shift slightly towards a lower binding energy (approximately 0.2 eV) with an increasing annealing temperature. This redshift is consistent with the conversion of Cr^3+^ into Cr^4+^ [20, 21].

### The Optical Property of the Samples

**Fig. 4:**
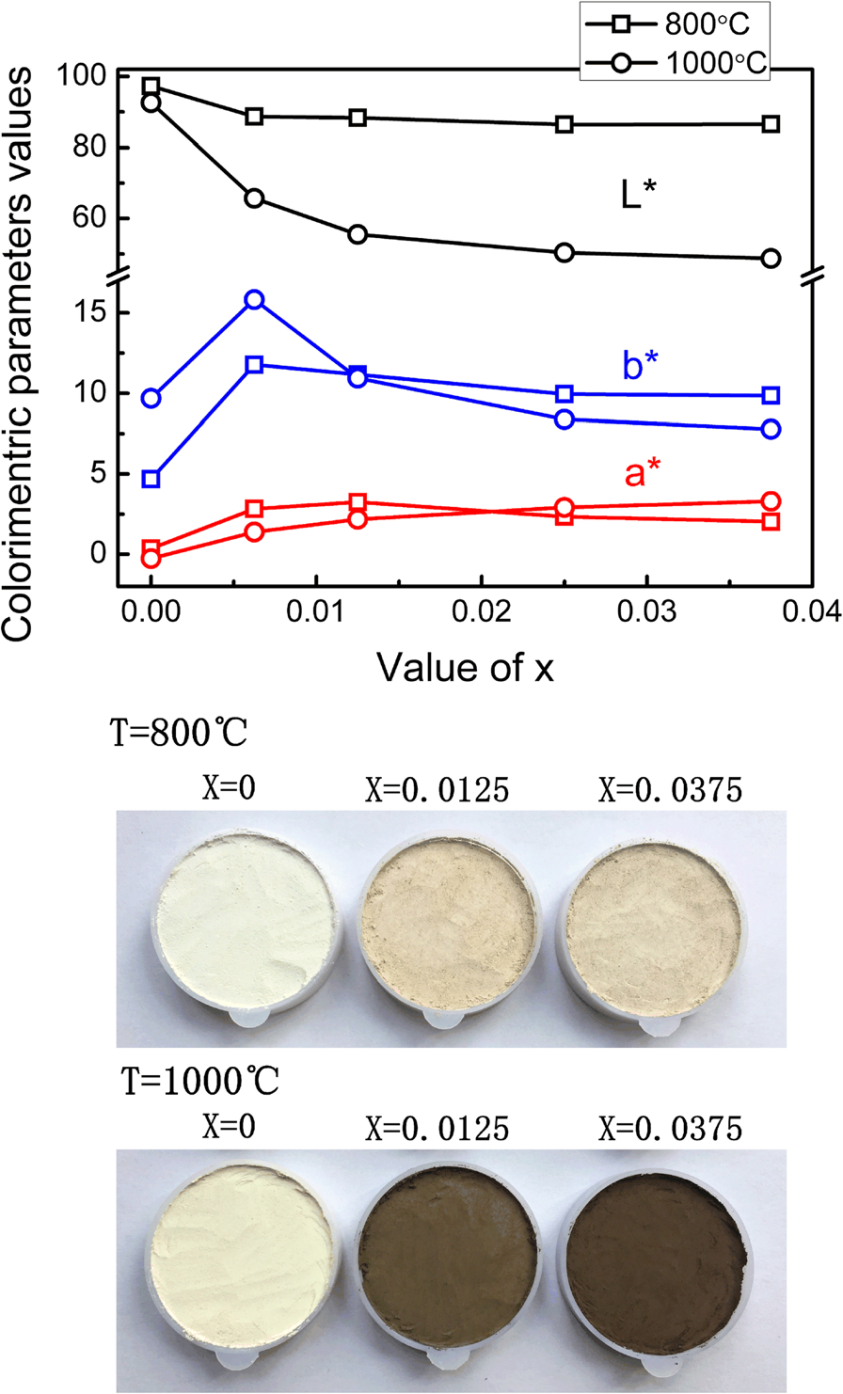
Color (CIE *L* ^*^ *a* ^*^ *b* ^*^) of Ti_1 − *x*_Cr_*x*_O_2_ pigments with various sintering temperatures and Cr concentrations

Figure 4 shows the colorimetric values of Ti_1 − *x*_Cr_*x*_O_2_ pigments with different sintering temperatures and doping concentrations. For the samples obtained at 800 °C, the variation in luminosity (*L* ^*^) is negligible as the dopant content increased. Meanwhile, the red component (*a* ^*^) and yellow component (*b* ^*^) first increase and then decrease with the increasing concentration of the Cr^3+^ impurity. Thus, the color of as-prepared anatase pigments changed from the original white into a brownish-yellow color.

When the sintering temperature increases to 1000 °C, the variations in *L* ^*^ and *b* ^*^ are more pronounced. As the Cr dopant content increases from *x* = 0 to 0.0375, the value of *L* ^*^ and *b* ^*^ decreases by 43.9 and 1.9, respectively. However, the change in *a* ^*^ is not the same as that of anatase samples that increase monotonously with the increasing Cr concentration. In the rutile Ti_1 − *x*_Cr_*x*_O_2_ pigments, the color changed remarkably from pale yellow to black brown, and the visible brightness was significantly inhibited. Thus, the Cr dopant can effectively modulate the color of rutile pigments, but there is little change on the anatase samples. The differential impact of Cr-doping on color properties is caused by the differences in the visible light reflectance spectrum. A lower visible reflectance results in more absorbed photons and a deeper color.

**Fig. 5:**
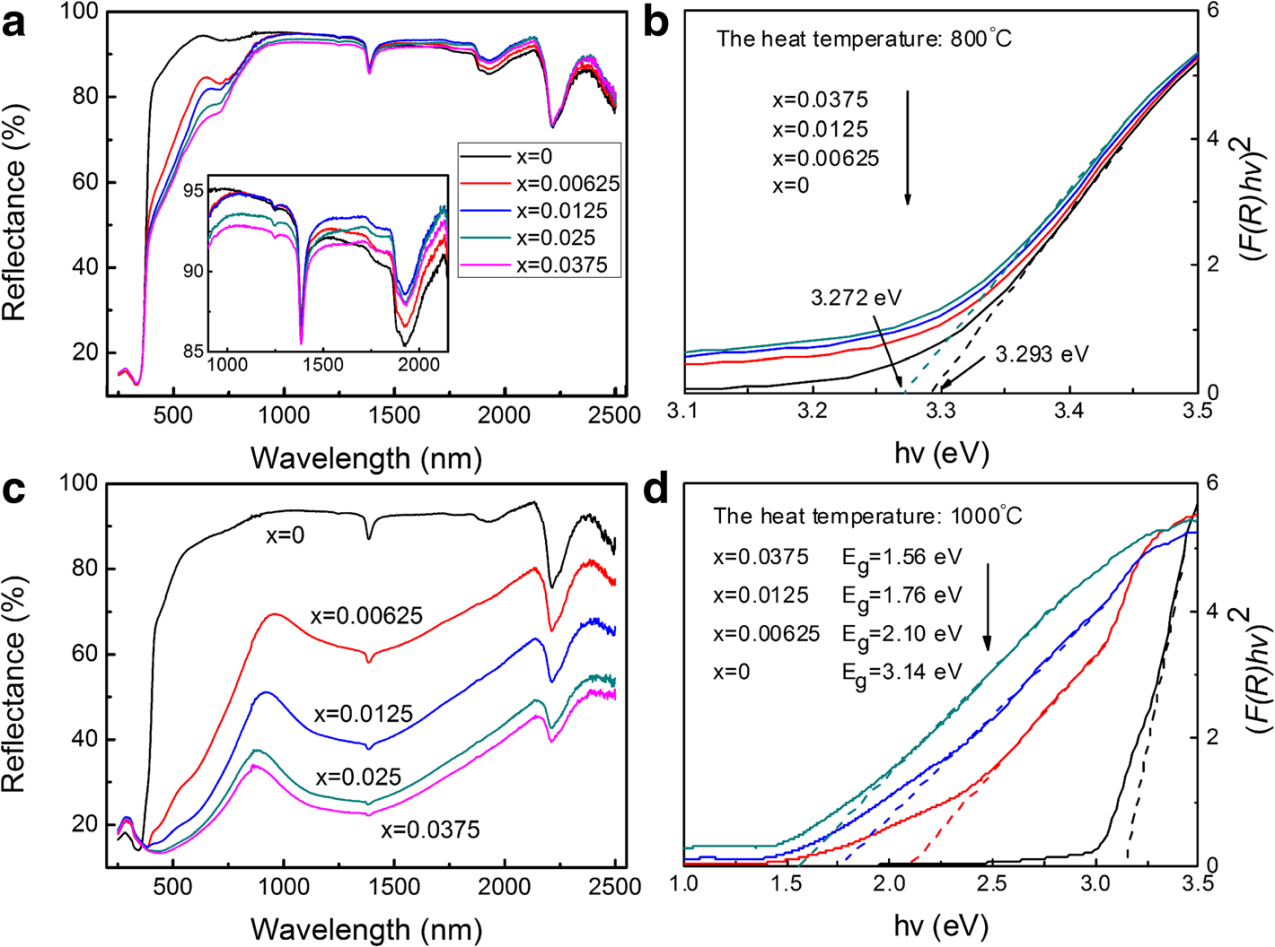
The UV-VIS-NIR diffuse reflectance spectra and *E*_*g*_ of Ti_1 − *x*_Cr_*x*_O_2_ samples with different sintering temperatures and Cr concentrations (**a**, **c** the raw data; **b**, **d** Kubelka-Munk transformed reflectance spectra)

**Fig. 6:**
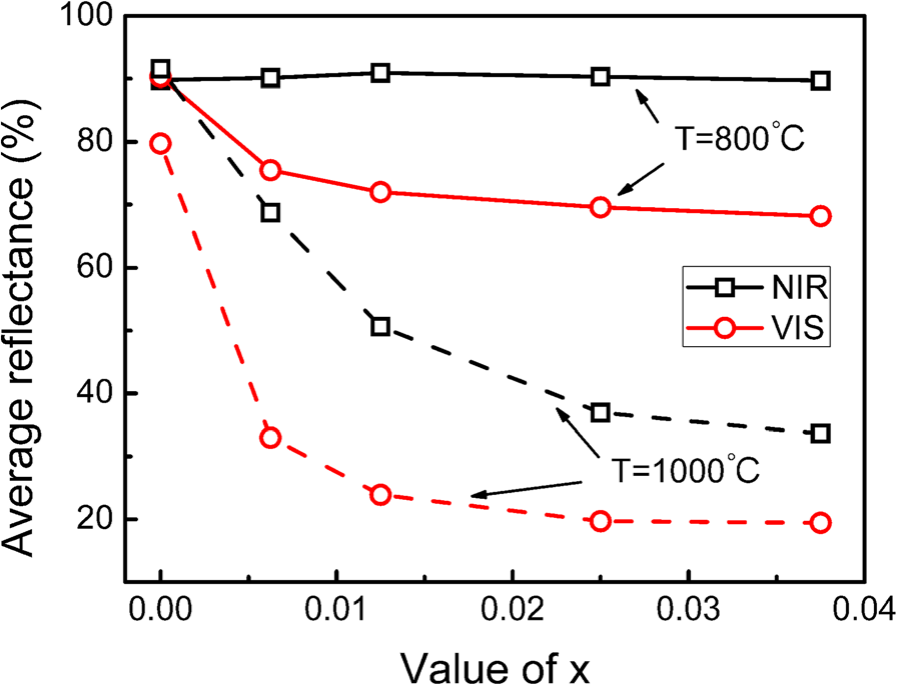
Effect of Cr concentration on the average spectral reflectivity of Ti_1 − *x*_Cr_*x*_O_2_ samples (VIS, 0.4–0.8 μm; NIR, 0.8–2.5 μm)

Figure 5 shows the UV-VIS-NIR diffuse reflectance spectra of undoped TiO_2_ and Ti_1 − *x*_Cr_*x*_O_2_ products with different sintering temperatures and Cr concentrations. Figure 6 shows the average spectral reflectivity of samples in the VIS (0.4–0.8 μm) and NIR (0.8–2.5 μm) ranges, respectively. The absorption peaks at 1384, 1926, and 2210 nm are attributable to the testing equipment and fixture in the spectra curves. Figures 5 and 6 show that the undoped TiO_2_ samples, whether anatase or rutile, have extremely high spectral reflectance in their near-infrared waveband (~ 90%). As the crystal phase transitions from anatase to rutile, its visible reflectance is still more than 80% even though the VIS absorption increased slightly.

For the Cr-doped anatase TiO_2_ sample, some extra absorption peaks can be detected in the visible light reflection cure. The VIS absorption peak at ~710 nm is related to the d-d electronic transition of Cr^3+^ in the octahedral crystal field of TiO_2_ [22], which could be assigned to the ^4^A_2_(F) → ^2^E electronic spin allowed transitions of Cr^3+^ [17]. At higher Cr^3+^ concentrations, there is stronger intensity absorption in the VIS waveband. Thus, the average VIS reflectance declines from 90.3% (*x* = 0) to 68.2% (*x* = 0.0375). Although the VIS reflectivity spectra are somewhat inhibited, the samples can maintain high reflectance in the near-infrared waveband (~ 90%).

When the sintering temperature increases to 1000 °C, the rutile phase TiO_2_ are finally transformed by anatase phase TiO_2_ in the Cr-doped products according to the XRD data. Figure 5c indicates two new absorption shoulders located at 450 and 600 nm in the rutile TiO_2_ samples. In particular, a strong and broad absorption band appeared in the near-infrared spectrum (about 1150 ~ 1500 nm). This is attributed to the ^3^A_2_ → ^3^ T_1_ electric dipole-allowed transitions of Cr^4+^ in the tetrahedral coordination [23, 24]. The absorption intensity gradually enhances with increasing dopant concentration.

In addition, the absorption edge of the rutile Ti_1 − *x*_Cr_*x*_O_2_ samples has an obvious redshift. However, there is no significant change in the absorption edge of the anatase samples. The diffuse reflectance spectra of the samples after Kubelka-Munk treatment are shown in Fig. 5b, d. The intersection between the linear fit and the photon energy axis gives the value to *Eg*. The relationship of band gap energy with absorption edge (*E*_*g*_ = 1240/*λ*_*g*_) suggests that the redshift of the absorption edge indicates a decrease in the band gap. Figure 5b shows that the doping process would not significantly alter the value of *E*_*g*_ for the anatase samples. This adds only 0.021 eV with the Cr content that increases to *x* = 0.0375. In contrast, the *E*_*g*_ value of rutile Ti_1 − *x*_Cr_*x*_O_2_ samples dropped sharply with the increasing doping concentration. The band gap reduces to 1.56 eV when the doping concentration is *x* = 0.0375.

In conclusion, the influence of Cr dopants on the spectral characteristic of TiO_2_ depends significantly on the crystal structure of the host materials. After introducing Cr dopant into the anatase TiO_2_ sample, only some characteristic absorption peaks appear in the visible light waveband due to the formation of an impurity energy level, while the band gap and NIR reflectance are not significantly affected. Thus, the near-infrared reflectance of anatase Ti_1 − *x*_Cr_*x*_O_2_ pigments remains at 90%. In rutile TiO_2_, however, the doping process leads to strong characteristic absorption peaks in both the VIS and NIR region. In addition, the decrease in band gap, *E*_*g*_, results in the enhanced capability to absorb lower energy photons. The VIS and NIR average reflectance of the rutile Ti_1 − *x*_Cr_*x*_O_2_ sample decreases by 60.2 and 58%, respectively, as the Cr content increases from *x* = 0 to 0.0375.

## Conclusions

We conclude that the crystalline phase, morphology, and optical properties of Ti_1 − *x*_Cr_*x*_O_2_ pigments are obviously affected by the sintering temperature and Cr-doped concentration. The incorporation of Cr^3+^ can accelerate the transition from anatase phase to rutile phase and compress the crystal lattice resulting in a 100 °C decrease in phase transition temperature. The doped ions rarely affect the morphology of anatase samples, but greatly increase the particle size and morphology of the rutile samples. This changes the morphology of rutile particles from columnar to near spherical at high doping concentrations.

Furthermore, the doping ions and crystalline structure have an important influence on the energy gap and optical properties of Ti_1 − *x*_Cr_*x*_O_2_ pigments. Cr^3+^ is gradually oxidized to Cr^4+^ during high-temperature sintering, and the Cr^4+^ content is greater as the sintering temperature increases. The generated Cr^4+^ ions lead to a strong NIR absorption band for rutile samples due to the ^3^A_2_ → ^3^ T_1_ electric dipole-allowed transitions of Cr^4+^. Furthermore, the band gap values of the rutile samples decreased gradually, and its absorption edges exhibited an obvious redshift as the doping concentration increased. This greatly enhanced the capability to absorb lower energy photons. Thus, the visible color changes to black brown as the Cr content increases from *x* = 0 to 0.0375. The VIS and NIR average reflectance of the rutile Ti_1 − *x*_Cr_*x*_O_2_ sample decreases by 60.2 and 58%, respectively.

Conversely, the anatase samples have only some characteristic absorption peaks that appear in the VIS waveband due to the formation of the impurity energy level of Cr^3+^. However, the band gap and NIR reflectance are not significantly affected. Thus, Cr-doped anatase TiO_2_ pigment with a brownish-yellow color and 90% near-infrared reflectance were obtained through this process.

## Abbreviations

*a*^***^: the CIE red component
*b*^***^: the CIE yellow component
*L*^***^: the CIE luminosity
NIR: Near infrared
UV: Ultraviolet
VIS: Visible light
